# Assessment of transfusion-induced iron overload with T2*MRI in survivors of childhood acute lymphoblastic leukemia: A case control study

**DOI:** 10.1016/j.htct.2024.09.2478

**Published:** 2024-10-18

**Authors:** Laila M. Sherief, Mohamed Beshir, Sahar N Saleem, Wesam Elmozy, Mona Elkalioubie, Basma K Soliman, Amr M Fawzy, Mona Alsharkawy, Diana Hanna

**Affiliations:** a Faculty of Medicine, Zagazig University, Egypt; b Kasr Al Ainy Faculty of medicine, Cairo University, Egypt

**Keywords:** Acute lymphoblastic leukemia survivors, Iron overload, Liver T2* MRI

## Abstract

**Introduction:**

Childhood acute lymphoblastic leukemia survivors receiving multiple packed red blood transfusions may be at risk of vital organ iron deposition causing long-term complications. This study was undertaken to assess the prevalence and severity of iron overload in the liver and heart by magnetic resonance imaging.

**Methods:**

A case-control study was conducted on 60 acute lymphoblastic leukemia survivors aged from 6 to 18 years and 60 healthy, age- and sex-matched children as a control group. The hematological profile, and serum ferritin was assessed and the iron content of the liver and heart was measured by T2* magnetic resonance imaging.

**Results:**

Twenty-six (43.3 %) and two (3.3 %) patients had elevated liver and myocardial iron concentrations, respectively. The statistics show a significantly positive correlation between liver T2* magnetic resonance and serum ferritin. The total volume of blood transfused and duration of follow up were associated with elevated liver iron concentrations (*p*-values = 0.036 and 0.028 respectively). Myocardial T2* magnetic resonance lacked correlation with serum ferritin and transfusion therapy

**Conclusion:**

Liver iron overload was detected in children and adolescents after acute lymphoblastic leukemia therapy. The risk of iron overload was related mainly to the transfusion burden during therapy. These patients need monitoring after therapy to assess their need for future chelation therapy.

## Introduction

Acute lymphoblastic leukemia (ALL) is the most common childhood cancer, with a reported prevalence of 25 % of cancers in under 15-year-old children [1]. Myelosuppressive chemotherapy protocols utilized in childhood ALL necessitate frequent red blood cell (RBC) transfusions which put patients at risk of iron overload and its long-term complications like cardiomyopathy and hepatic failure [2]. Iron overload contributes to increased morbidity in ALL survivors, who are already at risk of cardiac and hepatic dysfunction due to the late effects of chemotherapy [3].

Preliminary evidence for iron overload in pediatric oncology patients has been documented, however, its prevalence, organ distribution, and severity require further characterization [4]. Historically, direct measurement of tissue iron has been limited to liver biopsy. Magnetic resonance imaging (MRI) has emerged as an accurate, noninvasive and widely available tool for measuring iron in multiple organ systems [5]. R2 and R2* MRI relaxometry (R2  =  1/T2, R2*  =  1/T2*) have emerged as reliable alternatives to liver biopsy to monitor body iron content as correlations of a biopsy with proven liver iron content have been observed [6,7].

This technique is simple, allowing rapid and reproducible quantification of myocardial and hepatic iron and therefore allows preclinical detection of myocardial hemosiderosis. The T2* technique has the potential to become a noninvasive standard technique for tissue iron assessment [8].

We hypothesized that child and adolescent ALL survivors would develop iron overload due to blood transfusions previously received during the course of therapy. This study was conducted to assess the frequency, and severity of iron overload in the liver and heart as well as to measure serum ferritin in pediatric ALL survivors by means of cardiac and liver MRI.

## Material and methods

This case-control study was conducted on 60 pediatric ALL survivors in two tertiary care pediatric oncology centers, the Hematology and Oncology Department of the Children's Hospital, Zagazig University and Banha specialized children's hospital. Sequential eligible patients from regular follow up consultations at the oncology clinics from January 2018 to October 2018 were enrolled. Sixty healthy, age- and sex-matched controls were recruited from the community. We tried to match controls to cases for baseline characteristics like age, sex and place of residence.

This study was approved by the Institutional Review Board (IRB) of the Faculty of Medicine, Zagazig University and legal guardians signed an informed consent form before enrollment in the study.

ALL survivors of both sexes who were under 18-years old at the time of ALL diagnosis and had received a minimum of five packed RBC (PRBC) transfusions during the course of therapy were included if they had completed from 1 to 2 years of follow-up after treatment.

Patients were excluded if they were still under treatment for ALL and if they had malignancies other than ALL as were patients who had organ impairment (liver, kidney, heart). Moreover, patients with personal or familial history of hemochromatosis or hemoglobinopathies were excluded.

Clinical and treatment data were extracted from electronic and paper medical records. Data collected included age at treatment initiation and at time of the study, duration of follow up, and mean volume of blood transfusions received.

Serum ferritin was measured and echocardiography and liver and heart T2* MRIs were performed.

### Laboratory

Complete blood count (CBC) was performed on a Sysmex KX-21 Hematology Analyzer with examination of Leishman-stained peripheral blood smears. Assessments of liver and renal function tests were achieved using a Dimension Chemical auto analyzer.

Serum ferritin was measured using a human enzyme-linked immunosorbent assay (ELISA) kit (Thermo Fisher, Vilnius, Lithuania). Color changes were detected at a wavelength of 450 nm using an ELISA microplate reader 2100 Stat Fax (Awareness Technology. Inc., USA) based on the instructions provided by the manufacturer.

Hepatitis C virus (HCV) was assessed by quantitative real time polymerase chain reaction (PCR) using a COBAS amplicor 2.0 equipment (Roche Molecular Diagnostics, Pleasanton, CA, USA) with a lower limit of detection of 10 IU/mL.

### Imaging

#### Echocardiography

All studied patients were clinically asymptomatic for cardiovascular abnormalities. Cardiac measurements were performed according to the guidelines of the American Society of echocardiography [9].

### T2* magnetic resonance imaging

A 1.5 Tesla Siemens MRI machine (MAGNETOM Aera, Erlangen, Germane) was used for all patients. All MRI images were obtained without contrast and using a four-element phased-array coil. For the cardiac MRI, localizer sequences were followed by assessment of ventricular function via steady-state free-precession (SSFP) sequences in short-axis, two-chamber and four-chamber views and displayed as a cine loop format. A single breath-hold multi-echo bright blood sequence was used for the generation of cardiac T2* images of the entire studied group. The parameters for this sequence were: nine different echo times, TEs ranging from 1.88 to 15.4 ms, 10 mm slice thickness, 20-degree flip angle, in-plane spatial resolution 2.34 × 1.56 mm, field-of-view 400 × 300 mm and sampling bandwidth of 810 Hz/pixel [10].

A single axial mid-hepatic multiecho fast gradient-echo T2* sequence was performed for the liver. A single breath-hold sequence was used for all the studied group to obtain eight images with TEs ranging from 1.6 to 14 ms, 10 mm slice thickness, 20-degree flip angle, in-plane spatial resolution of 2.7 × 3.1 mm, field-of-view 350×250 mm and sampling bandwidth of 125 Hz/pixel. Post processing image analysis and measurement of T2* was done on the Siemens MMWP Multi-Modalities workstation. The measurement process comprised importing the image to the program's database, drawing a region of interest (ROI), then applying tools to calculate T2* with motion correction, proper fitting, and writing final values [10].

For the heart, a ROI was drawn through the full thickness of the septum wall of the myocardial short axis image. The ROI was chosen to include both endocardium and epicardium as well as septum from both ventricular intersections. Mean size of ROI was 2.75 ± 0.81 cm^2^.

For the liver, a ROI was drawn within the right liver lobe on T2* maps, excluding any vasculature identified visually: mean ROI size was 18.4 ± 6.72 cm^2^.

### Image analysis

Two radiologists with ten years cardiac MRI experience, independently assessed the cardiac SA and liver axial T2* parametric maps. The readers were blinded for clinical information, including reason for referral.

The T2*, R2*, and liver iron concentration (LIC) were automatically calculated from the datasets included in the analyses. Heart and liver plot charts were generated with results being represented in a colored manner based on the severity of iron burden: green for normal values, mild affection was presented in yellow, moderate in orange, and severe in red.

According to calculated T2*, cardiac iron load was classified as acceptable (T2*: >20 ms), mild (T2*: 15–20 ms), moderate (T2*: 10–14 ms), and severe (T2*: <10 ms).

Regarding the liver T2* values were normal >15.4 ms, mild 4.5–15.4 ms, moderate 2.1–4.5 ms and severe <2.1 ms[10].

R2* Hz (1000/T2* ms), liver R2* values was classified as normal <65 Hz, mild 65–222.2 Hz, moderate 222.2–476.2 Hz and severe >476.2 Hz.

LIC was calculated using the Garbowski equation [11] (LIC (mg g^-1^) = 0.032R2*- 0.14) where 2–7mg g^-1^ dry liver weight indicates light, 7–15 mg g^-1^ dry weight indicates moderate and >15 mg g^-1^ dry weight indicates severe iron burden.

### Statistical analysis

The collected data were tabulated and analyzed using the Statistical Package for Social Sciences (SPSS) version 24 software (SPSS Inc, Chicago, IL USA). Categorical data are presented as number and percentages while quantitative data are expressed as mean ± standard deviation, median and range. The Chi-square test (χ[2]) or Fisher's exact test were used to analyze categorical variables. Quantitative data were tested for normality using the Kolmogorov-Smirnova test, assuming normality for p-values >0.05. The Student *t*-test was used to analyze normally distributed variables between two independent groups. While non-parametric variables were analyzed using the Mann-Whitney U test. Receiver operating characteristic (ROC) curves were used to determine the best cutoff value for ferritin and volume of RBCs transfused with best combined sensitivity and specificity. The area under the curve (AUC) was calculated for each plot. The significance of the results was determined using *p*-values ≤0.05.

## Results

This study included 60 ALL survivors; 32 were female (53.3 %) and 28 were male (46.7 %). The mean ± standard deviation (SD) of the age at the time of study was 11.83 ± 3.51 years, age at diagnosis was 6.66 ± 3.53 years and duration of follow up was 1.46 ± 0.448 years. Forty-four patients (73.3 %) were diagnosed with precursor B-cell ALL (73.3 %) and 16 patients (26.7 %) were diagnosed with T-cell ALL.

The majority of patients (73.3 %) received standard-risk therapy regimens (according to the Children's Cancer Group - CCG protocol), while 26.7 % of the patients received high-risk protocols. Patients received mean volume of 6256.66 ± 2401.82 mL of transfused PRBCs (range: 143.8 ± 45.5 mL/kg). None of the enrolled patients had received iron chelation therapy. Forty patients (66.7 %) were positive for hepatitis C markers.

There were statistically lower hemoglobin and platelet levels in the Patient Group, while higher alanine transaminase (ALT) and aspartate transaminase (AST) were detected in patients when compared with controls. Significantly higher serum ferritin levels were detected in patients (*p*-value = 0.000). The demographics and laboratory features of the groups are summarized in Table 1.

**Table 1:** Comparison between pediatric ALL survivors and controls regarding laboratory parameters.

Variable	Patients (*n* = 60)	Controls (*n* = 60)	*p*- value
	Mean ± SD	Mean ± SD	
Age	11.83 ± 3.51	11.54 ± 4.11	0.678
Sex: Male - n (%)	28 (46.7)	25 (41.7)	
Female - n (%)	32 (53.3)	35 (58.3)	
Follow up duration (years)	1.46 + 0.448	–	–
Total volume of transfused RBCs (mL)	6256.66 ± 2401.82	–	–
Transfused RBCs (mL/kg)	143.8 ± 45.5	–	–
Weight (kg)	41.86 ± 9.45	42.70 ± 12.91	0.777
Height (cm)	150.60 ± 9.84	147.30 ± 13.91	0.293
BMI	18.37 ± 3.08	19.03 ± 2.72	0.383
Hb (g/dL)	11.70 ± 0.979	12.38 ± 0.418	0.000
TLC (10^9^ cells/L)	7.71 ± 1.77	7.32 ± 1.39	0.182
PLT (10^9^ cells/L)	239.90 ± 63.19	290 ± 41.24	0.000
Neutrophils (10^9^ cells/L)	4.23 ± 1.13	4.15 ± 0.762	0.650
ALT (u/L)	43.77 ± 34.26	20 ± 5.38	0.000
AST (u/L)	51.7 ± 42.92	25.40 ± 5.64	0.000
Albumin (g/dL)	4.03 ± 0.538	4.26 ± 0.417	0.010
TSB (mg/dL)	0.870 ± 0.093	0.873 ± 0.103	0.867
DSB (mg/dL)	0.181 ± 0.048	0.117 ± 0.022	0.000
Urea (mg/dL)	32.76 ± 5.67	32.70 ± 2.57	0.941
Creatinine (mg/dL)	0.570 ± 0.120	0.500 ± 0.143	0.004
Serum ferritin level (µg/L)	447.54 ± 434.20	100.55 ± 4.30	0.000

Hb, hemoglobin; SD, standard deviation; TLC, total leukocyte count; PLT, platelet count; ALT, alanine transaminase; AST, aspartate transaminase; TSB, total serum bilirubin; DSB, direct serum bilirubin; <.05: significant; >.05: not significant.

There were no statistically significant differences in respect to the cardiovascular (systolic blood pressure, diastolic blood pressure, and heart rate) and echocardiography parameters between patients and controls, as shown in Table 2.

**Table 2:** Comparison between patients and controls regarding cardiovascular and echocardiography parameters.

Variable	Patients (*n* = 60)	Controls (*n* = 60)	*p*- value
	Mean ± SD	Mean ± SD	
Systolic blood Pressure (mmHg)	106.06 ± 3.66	105.50 ± 4.79	0.473
Diastolic blood Pressure (mmHg)	65.80 ± 5.36	64.50 ± 5.77	0.204
Heart rate (BPM)	79.80 ± 6.29	80.70 ± 4.59	0.373
EF (%)	72 ± 4	72 ± 2	1.00
FS (%)	36 ± 3	36 ± 3	1.00
LVES (mm)	25.36 ± 2.90	25 ± 3.26	0.524
LVED (mm)	37.40 ± 2.84	37.50 ± 3.02	0.852
IVS (mm)	7.30 ± 0.545	7.18 ± 0.630	0.267

BPM, beat per minute; EF, ejection fraction; FS, fraction shortening; LVES, left ventricular end systolic; LVED, left ventricular end diastolic; IVS, Interventricular septum.

Regarding the liver T2*and R2* images and LIC, 23.3 % of the patients showed mild hepatic iron overload and 13.3 % showed severe hepatic iron overload; moderate hepatic overload presented in only 6.7 % while 56.7 % had normal hepatic iron loads (Table 3). While in the heart T2* images, most of patients showed normal iron loads (96.7 %) with only two patients having moderate iron overload (3.3 %).

**Table 3:** Distribution of liver T2* magnetic resonance imaging of pediatric ALL survivors.

Liver iron overload	Patient		T2*Liver (ms)	R2*Liver (Hertz)	LIC (mg g^-1^)
	n	%			
Normal	34	56.7	28.43 ± 6.8	37.1 ± 9.01	1.8 ± 0.17
Abnormal	26	43.3	7.2 ± 5.2	406.1 ± 566.6	12.8 ± 16.9
Mild	14	23.3	11.75 ± 1.7	86.8 ± 13.02	3.3 ± 0.3
Moderate	4	6.7	3.01 ± 0.77	343.6 ± 71.8	10.9 ± 2.1
Severe	8	13.3	1.38 ± 0.67	996.1 ± 734.8	30.5 ± 22.04
Total	60	100.0	19.24 ± 12.3	197.03 ± 412.3	6.6 ± 12.3

Liver T2* values: normal >15.4 ms; mild from 4.5 to 15.4 ms; moderate from 2.1 to 4.5 ms; severe <2.1 ms.

Liver R2* values: normal < 65 Hz; mild from 65 to 222.2 Hz; moderate from 222.2 to 476.2 Hz; severe >476.2 Hz.

Liver iron concentration (LIC): normal < 2 mg g^-1^, mild 2–7 mg g^-1^, moderate 7–15 mg g^-1^ and severe >15 mg g^-1^. dry weight liver.

The liver T2* MRI showed significantly higher levels among ALL survivors with negative hepatitis C markers (*p*-value = 0.004) and in those who received higher mean volumes of blood transfusion (*p*-value = 0.036), and who had higher serum ferritin levels (*p*-value = 0.000; Table 4).

**Table 4:** Relation between liver T2* magnetic resonance imaging and different parameters of pediatric ALL survivors.

Variable		Normal T2* liver		Abnormal T2* liver	*p*-value
Hepatitis C markers	Positive	**n**	30	10	0.004
		**%**	88.2 %	38.5 %	
	Negative	**n**	4	16	
		**%**	11.8 %	61.5 %	
Follow up duration	Mean ± SD	1.62 ± 0.452		1.26 ± 0.366	0.028
Volume of blood transfusion (cc)		5905.88 ± 2265.63		6715.38 ± 2587.42	0.036
ALT (u/L)		40.61 ± 27.94		46.18 ± 39.08	0.667
AST (u/L)		48.30 ± 31.14		54.29 ± 50.95	0.712
Albumin (g/dL)		4.11 ± 0.591		3.91 ± 0.456	0.316
TSB (mg/dL)		0.861 ± 0.108		0.882 ± 0.070	0.559
DSB (mg/dL)		0.181 ± 0.049		0.181 ± 0.048	0.990
Urea (mg/dL)		32.05 ± 6.66		33.69 ± 4.11	0.444
Creatinine (mg/dL)		0.588 ± 0.126		0.546 ± 0.112	0.353
Serum ferritin level (µg/L)		188.60 ± 179.30		786.15 ± 439.95	0.000

ALT, alanine transaminase; AST, aspartate transaminase; TSB, total serum bilirubin; DSB, direct serum bilirubin.

Normal T2* liver: >15.4 ms; Abnormal T2* liver: ≤ 15.4 ms.

There was a significant positive correlations of T2* MRI images of the liver with age of patients (*p*-value = 0.002), mean volume of blood transfusion (*p*-value = 0.036) and serum ferritin levels (*p*-value = 0.000) as shown in Table 5.

**Table 5:** Correlation between liver T2* magnetic resonance imaging and different parameters of pediatric ALL survivors.

Variable	T2* liver	
	R	*p*-value
Serum ferritin level	0.705	0.000
ALT	0.127	0.504
AST	0.088	0.642
Age	0.54	0.002
Mean volume of transfused blood	0.812	0.036

ALT, alanine transaminase; AST, aspartate transaminase.

**Figure 1:**
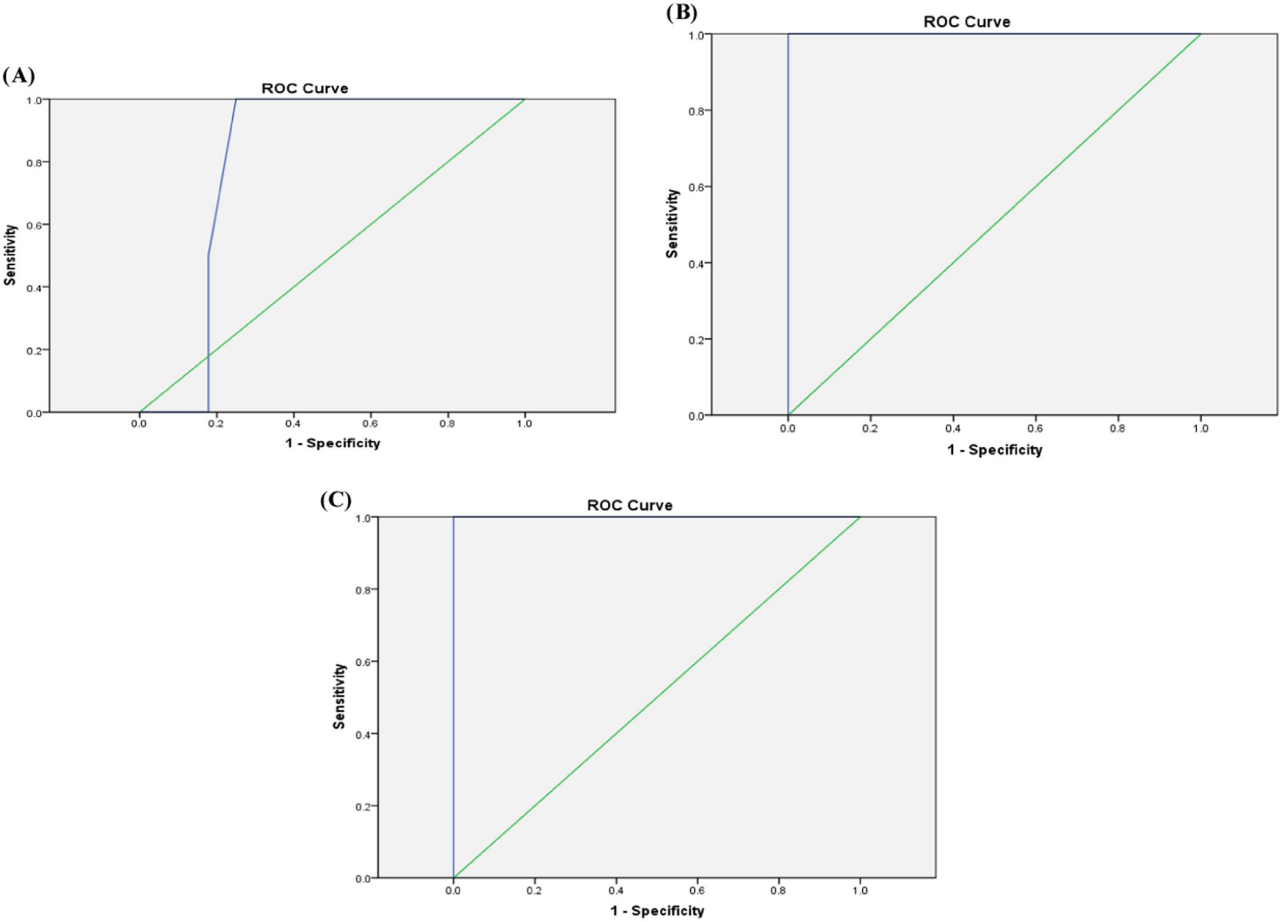
Receiver operator curve (ROC) showing cutoff points for serum ferritin to detect mild (1A), moderate (1B), severe (1C) liver iron overload by liver T2* magnetic resonance imaging in pediatric ALL survivors.

ROC analysis revealed that a cutoff value for serum ferritin of 205 µg/L could be used as a predictor for the presence of mild liver iron overload among ALL survivors with a sensitivity of 71.4 %, specificity of 66 %, accuracy of 66.7 %, and AUC: 0.643 (*p*-value = 0.108-Figure 1A). A value ≥725 µg/L and <850 µg/L would detect moderate liver iron overload with sensitivity of 100 %, specificity of 75 %, accuracy of 76.7 % and AUC: 0.804 (*p*-value = 0.04-Figure 1B). A cutoff value of 850 µg/L would identify severe liver iron overload with a sensitivity of 100 %, specificity of 96.2 %, accuracy of 96.7 % and AUC: 1.00 (*p*-value <0.001-Figure 1C).

**Figure 2:**
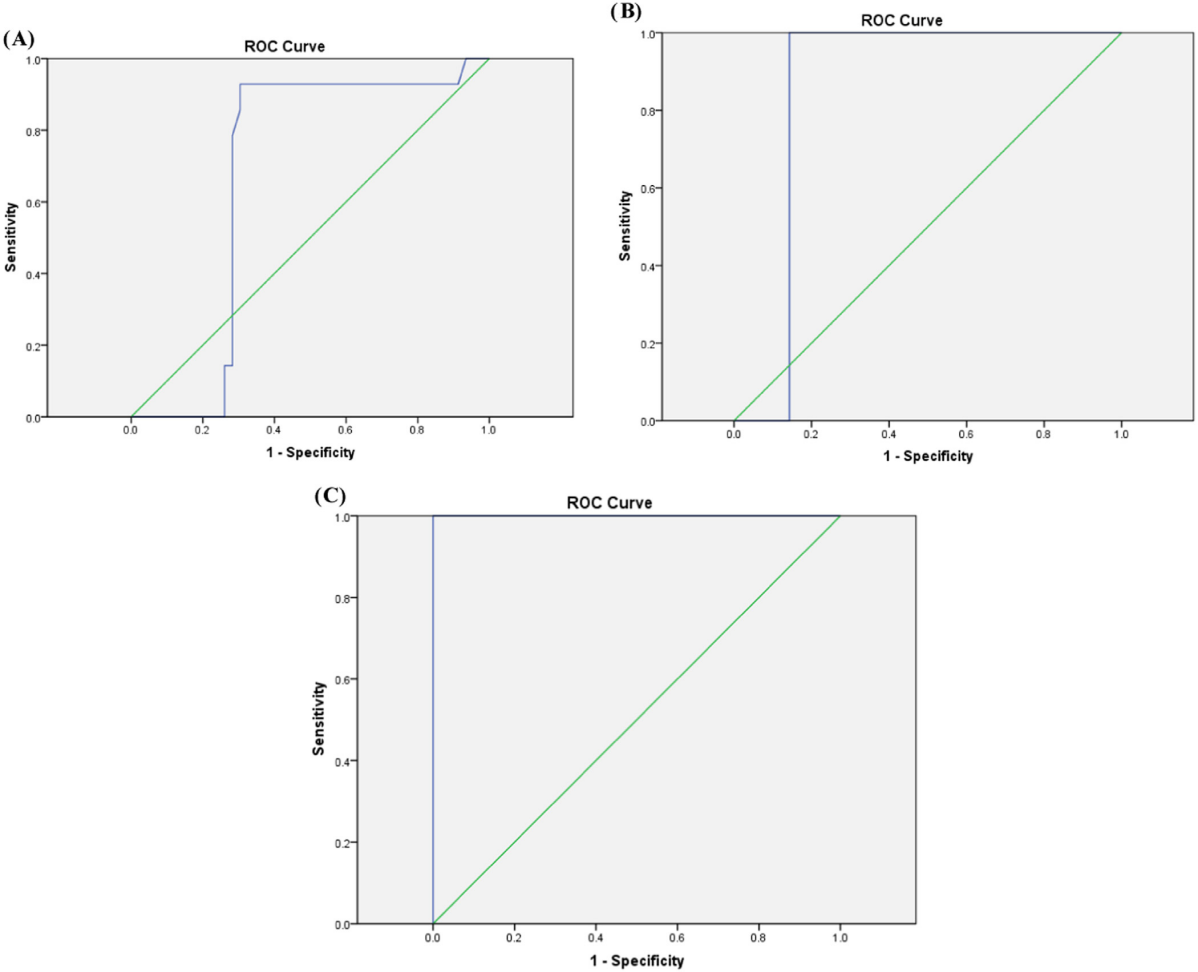
Receiver operator curve (ROC) showing cutoff points for amount of transfused blood in relation to weight (mL/kg) to detect mild (2A), moderate (2B), severe (2C) liver iron overload in pediatric ALL survivors.

Regarding volume of blood transfusion/kg, the means ± SD of 131.3 ± 21.4, 191 ± 5.11, and 217.17 ± 11.7 mL/kg were detected among the groups with mild, moderate, and severe liver iron overload, respectively according to T2* MRI. Moreover, a cutoff value of 115.1 mL/kg would detect mild liver iron overload with a sensitivity of 92.9 %, specificity of 70 %, accuracy of 75 % and AUC: 0.672 (*p*-value = 0.052-Figure 2A). A cutoff value of 169.4 mL/kg would detect moderate liver iron overload with sensitivity of 100 %, specificity of 86 %, accuracy of 86.7 % and AUC: 0.857 (*p*-value = 0.01-Figure 2B). Furthermore a cutoff value of 201.5 mL/kg would detect severe liver iron overload with a sensitivity of 87.5 %, specificity of 100 %, accuracy of 98.3 % and AUC: 1.00 (*p*-value <0.001-Figure 2C).

## Discussion

Iron overload is a significant cause of morbidity and mortality for patients who require frequent transfusions [12]. Thus, the recognition of iron overload is crucial in long-term survivors of childhood ALL [13]. Most studies on iron overload in cancer survivors have been among those diagnosed as adults and few studies have been conducted among survivors of childhood cancer [14]. The current study aimed to evaluate iron overload in survivors of childhood ALL using serum ferritin levels and T2* MRI as an accurate, non-invasive method for measuring iron in the liver and heart.

The current study showed that 43.3 % of ALL survivors (26 out of 60) had abnormal liver T2* MRI; almost half (14 out of 26) had mild iron overload, while severe and moderate iron loads were detected in eight and four patients, respectively. This was a slightly higher percent than that reported by Halonen et al. [15] who showed that iron overload in survivors of childhood cancer was detected in about 14 % of children recently treated for ALL. In addition, Ruccione et al. [13] reported elevated liver iron content in 36 out of 73 (49.3 %) childhood cancer survivors with one-third-of the study population having ALL (30.7 %) [13]. Nair et al. [3] also reported that one fourth of pediatric leukemia patients (16 out of 66) developed iron overload at the end of chemotherapy [3].

Serum ferritin levels were found to be significantly higher among patients compared to controls with 24 out of 60 patients (40 %) having high serum ferritin levels (>500 µg/L). Moreover, patients with liver iron overload detected by T2* MRI had higher serum ferritin levels than those without and a significant positive correlation was found between serum ferritin and T2* liver results (*p*-value = 0.000). Moreover, ROC analysis showed that cutoff values of serum ferritin of 205 µg/L, 725 µg/L, and 850 µg/L detected mild, moderate, and severe liver iron overload among ALL survivors.

In agreement with our results, many other studies showed a strong positive correlation between hepatic T2* MRI and serum ferritin levels [12,13,15, 16, 17, 18, 19]. Knovich et al. [20] concluded that ferritin concentration remains the most practical method for measuring total body iron [20]. However, some previous studies have failed to demonstrate a linear relationship between serum ferritin and hepatic iron concentration in diseases with transfusion-induced iron overload [21,22]**.** On the contrary, Fahmy et al. [22] detected a significant negative correlation between serum ferritin and liver T2* MRIs (*p*-value = 0.007), which coincides with the results of Fischer et al. [23] and Azarkeivan et al. [24] This indicates that serum ferritin levels cannot predict the liver iron content among patients [25]. This discrepancy may be explained by the fact that secondary factors such as infection, inflammation, and malignancy increase serum ferritin concentrations, and all these factors are occasionally present in acute leukemia. Moreover, leukemic blast cells are known to synthesize ferritin resulting in high pretreatment serum ferritin levels [26]. This may explain why ferritin levels were not correlated to liver T2* images at baseline. Nevertheless, since ferritin levels are correlated with liver MRI findings at one year of follow-up, serum ferritin level can be considered a good screening tool for liver iron overload in this population [27].

In the present study, a statistically significant positive correlation was demonstrated between the total volume of blood transfusions and iron overload in the liver (*p*-value = 0.036). ROC analysis showed that cutoff values for transfused PRBC volumes of 115.1 mL/kg, 169.4 mL/kg, and 201.5 mL/kg would detect mild, moderate, and severe liver iron overload, respectively.

This finding is consistent with other studies which reported that cumulative PRBC transfusions were positively associated with MRI findings of liver iron overload [9,17,25].

Munikoty et al. [28] reported that the number of RBC units transfused and duration from the last transfusion were associated with elevated serum ferritin (*p*-value  =  0.001 and *p*-value  =  0.002, respectively) and elevated LIC (*p*-value  =  0.012 and *p*-value  =  0.005, respectively) in multiple linear regression [28]. Ruccione et al. [13] suggested the potential value of tracking cumulative PRBC volume as a risk factor for iron overload, something that currently is not a standard practice [10]. Moreover, De Ville de Goyet et al. [27] recommended that all patients who received >1000 mL of PRBCs should be screened for iron overload with MRI regardless of the ferritin level [27].

Age is a complex factor in pediatric populations because there are many factors that affect the velocity of growth and development such as nutrition, pubertal status, and other factors [13]. This study revealed that there is a statistically positive correlation between age of the patients and iron overload detected by liver T2* MRI. Trovillion et al. [19] supported this observation, with all patients identified with iron overload being over 14 years of age at the time of diagnosis. Also, Amid et al. [4] observed that older age is associated with increased LIC [4]. Physical growth consumes iron stores, and therefore younger patients have more time to consume excess iron that may have accumulated during cancer treatment, while for adolescent patients, the growth phase is over, and therefore iron is stored throughout the body leading to potential complications [13].

In the current study, there was a statically significant difference between patients with normal T2*liver findings and patients with abnormal T2*liver findings regarding the follow up duration with the abnormal liver T2* measurements being higher in a group with shorter follow-ups (*p*-value = 0.028). The same relation was obtained by De Ville de Goyet et al. [27] who revealed that in the first year after treatment approximately 66 % of participants had liver iron overload, but after two years, this had already decreased to 48 % [27]. However, we think that further longitudinal studies are needed to evaluate the kinetics of the drop in serum ferritin from cessation of therapy and whether attainment of puberty after therapy hastens the decline in serum ferritin.

It is worth mentioning that 66.7 % of our patients had positive hepatitis C markers which may potentially cause liver injury and fibrosis. Although most HCV-positive patients (88.2 %) showed normal T2* liver images, further longitudinal studies are required to assess severity of liver injury and degree of liver fibrosis and also to investigate the role of iron overload and HCV positivity as independent risk factors for progression of hepatic fibrosis in pediatric ALL survivors.

Regarding heart T2* findings, moderate iron overload was detected in only two patients (3.3 %) and these patients had normal echo parameters. In agreement with our results, Munikoty et al. [28] reported that myocardial iron concentrations were observed in 4.5 % of children treated for hematological malignancies. Additionally, De Ville de Goyet et al. [27] observed that myocardial iron overload is present in 14 % of the cancer patients after one year of follow-up. However, some studies found no MRI evidence of cardiac iron overload even with liver iron overloading [13,29]. This can be explained by the fact that the liver is the dominant storage organ for excess iron, mobilizing iron rapidly and efficiently in times of demand. In contrast, the heart has robust mechanisms to prevent excess transferring-mediated uptake [30]. Even then, cardiac iron uptake is delayed compared to many other extra-hepatic organs. Thus many young patients show severe hepatic iron overloading with no evidence of cardiac iron overloading [31]. This explains why identification of abnormal systolic function is a late sign of myocardial iron toxicity. Iron clears more slowly from the heart than from the liver in response to iron chelation, which may contribute to the high mortality of patients with established cardiomyopathy [32].

On the other hand, Jensen et al. [33] found a significant positive linear relation between different iron store parameters and the MRI-derived myocardial iron concentration, which was significantly correlated to the serum ferritin concentration (*p*-value ˂0.0001) and to the MRI-determined liver iron concentration (*p*-value = 0.02) in 41 multiply transfused patients [33].

### Limitations

One major limitation in this study was the small sample size. In addition, patients did not represent all Egypt as they were from a limited geographical area. So, we still in need of larger multicenter studies to support these results. Another limitation is that we did not measure liver fibrosis in these patients; this will be considered in future research.

## Conclusion

In conclusion, liver iron overload was detected in children and adolescents after ALL therapy. The patients’ age, mean volume of blood transfusion, and serum ferritin levels were correlated with liver T2* MRI findings. There was no correlation between serum ferritin levels and myocardial T2* MRI. We recommend evaluation of iron status by serum parameters as serum ferritin and evaluation of liver and heart iron content using T2* MRI to better evaluate the state of iron overload in high-risk patients, allowing earlier chelation intensification with its potential to reduce adverse events. Large-scale longitudinal studies are still required to demonstrate the power of iron metabolism in cancer patients, challenging the importance of developing strategies to evaluate and measure the impact of iron accumulation in the body.

### Author contributions

Contributions to idea and design, L.M.S., M.B., S.N.S. and A.M.F.; methodology and acquisition of data, S.N.S., WE., M.E., A.M.F. and B.K.S.; analysis and interpretation of findings, L.M.S., M.B., S.N.S. and D.H.; writing original draft preparation, L.M.S. and D.H.; writing review and editing, L.M.S., M.B., S.N.S.; All authors have read and agreed to the published version of the manuscript.

## Conflicts of interest

The authors report no potential conflict of interests.
